# 3D Printing of Anisotropic Piezoresistive Pressure Sensors for Directional Force Perception

**DOI:** 10.1002/advs.202309607

**Published:** 2024-03-13

**Authors:** Jingfeng Liu, Xuan Zhang, Jintao Liu, Xingang Liu, Chuhong Zhang

**Affiliations:** ^1^ State Key Laboratory of Polymer Materials Engineering Polymer Research Institute of Sichuan University Chengdu 610065 China

**Keywords:** 3D printing, anisotropic pressure sensor, directional response, elastomeric conductive composite, tunable anisotropy

## Abstract

Anisotropic pressure sensors are gaining increasing attention for next‐generation wearable electronics and intelligent infrastructure owing to their sensitivity in identifying different directional forces. 3D printing technologies have unparalleled advantages in the design of anisotropic pressure sensors with customized 3D structures for realizing tunable anisotropy. 3D printing has demonstrated few successes in utilizing piezoelectric nanocomposites for anisotropic recognition. However, 3D‐printed anisotropic piezoresistive pressure sensors (PPSs) remain unexplored despite their convenience in saving the poling process. This study pioneers the development of an aqueous printable ink containing waterborne polyurethane elastomer. An anisotropic PPS featuring tailorable flexibility in macroscopic 3D structures and microscopic pore morphologies is created by adopting direct ink writing 3D printing technology. Consequently, the desired directional force perception is achieved by programming the printing schemes. Notably, the printed PPS demonstrated excellent deformability, with a relative sensitivity of 1.22 (kPa^*^wt. %)−1 over a substantial pressure range (2.8 to 8.1 kPa), approximately fivefold than that of a state‐of‐the‐art carbon‐based PPS. This study underscores the versatility of 3D printing in customizing highly sensitive anisotropic pressure sensors for advanced sensing applications that are difficult to achieve using conventional measures.

## Graphical abstract

An anisotropic piezoresistive pressure sensor (PPS) featuring tailorable flexibility in macroscopic 3D structures and microscopic pore morphologies is fabricated by adopting direct ink writing 3D printing technology. The optimized PPS can identify stress in different bending directions, which has attractive potential in wearable electronics, artificial intelligence, and soft robotic applications.

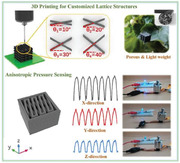

## 1 Introduction

The challenge of traditional pressure sensors in differentiating loading directions originates from the isotropic nature of sensing materials and structures, which typically generates similar deformations upon pressing in any direction.^[^ ^1^, ^2^ ^]^ Such features have severely limited their practical application when considering the multidimensionality of mechanical stimuli.^[^ ^3^ ^]^ To overcome these obstacles, anisotropic pressure sensors are necessary for recognizing complex pressure states accurately owing to their directional sensing behaviors, which have attractive potential in wearable electronics, artificial intelligence, and soft robotic applications.^[^ ^3^ ^–^ ^5^ ^]^

In recent years, a few anisotropic pressure sensors have been developed by constructing anisotropic sensing composites or structures to detect the loading directions. Pressure sensors with aligned sensing components, such as aligned graphite/silk fiber,^[^ ^6^ ^]^ carbon nanofibres,^[^ ^7^ ^]^ and polyvinylidene fluoride, also show different sensitivities parallel and perpendicular to the alignment direction. However, methods that rely on material modification suffer from complex synthetic processes and lack tailorable flexibility. Anisotropy can also be achieved by designing the anisotropic structure of the sensing units. Existing research focuses on directional pore channels, which typically generate varied deformations under pressure in given directions.^[^ ^8^, ^9^, ^10^ ^]^ For example, Wang et al.^[^ ^8^ ^]^ prepared a microcellular nanotube aerogel with unidirectionally arranged penetrating pore channels using a directional freeze‐drying technique, realizing the directional sensing recognition of pressure sensors in the axial and radial directions. However, these methods are complicated by the physical foaming process, and precisely controlling the pore channel structure is challenging, restricting the large directional sensitivity differences under different loading directions.

3D printing is an additive rapid prototyping technique that can easily and flexibly tailor arbitrary 3D structures on demand. It has gained considerable attention in the design of customized anisotropic architectures. Precisely constructing an anisotropic structure can ensure a clear distinction in the sensing sensitivity in any direction. However, only a few studies have constructed anisotropic piezoelectric 3D sensors through 3D printing. Cui et al.^[^ ^11^ ^]^ successfully reported a strategy for designing anisotropic smart infrastructures to manipulate a set of piezoelectric displacement maps with a given pressure, which could be leveraged to sense pressure from arbitrary directions. However, a few types of piezoelectric materials limit the selection of sensing materials. Moreover, piezoelectric devices with 3D structures typically suffer from poor polarisation efficiency when considering the thickness of 3D objects,^[^ ^12^, ^13^, ^14^ ^]^ which is detrimental to sensing performance. Piezoresistive pressure sensors (PPS) based on piezoresistive effects have a wider selection of conductive materials.^[^ ^15^, ^16^ ^]^ Unlike piezoelectric materials in piezoelectric devices, conductive agents in PPS do not require polarization. Therefore, the sensing performance of PPS is not affected by the polarization process, which is ideal for developing anisotropic 3D sensors. However, research on 3D anisotropic PPS by 3D printing is limited.

This study is the first to develop an innovative aqueous printable ink containing a waterborne polyurethane (WPU) elastomer. The ink was sculpted into arbitrary 3D shapes through direct ink writing (DIW) 3D printing technology. By simply adjusting the designed printing scheme, it was easy to regulate the sensitivity ratio in each direction and achieve a clear distinction in different directions. The unique anisotropic “scissor‐like” PPS could achieve the desired aimed functions, including pressure magnitude and directionality sensing without any additional sensing component. In addition, the printed PPS with macroscopic 3D structures and microscopic pore morphologies exhibited a high relative sensitivity. This work highlights 3D printing as a unique and powerful tool for modeling and manipulating piezoresistive regulable directional sensors, which has considerable application potential.

## 2 Results and Discussion

### 2.1 Preparation and Rheological Behaviors of Inks

**Figure 1:**
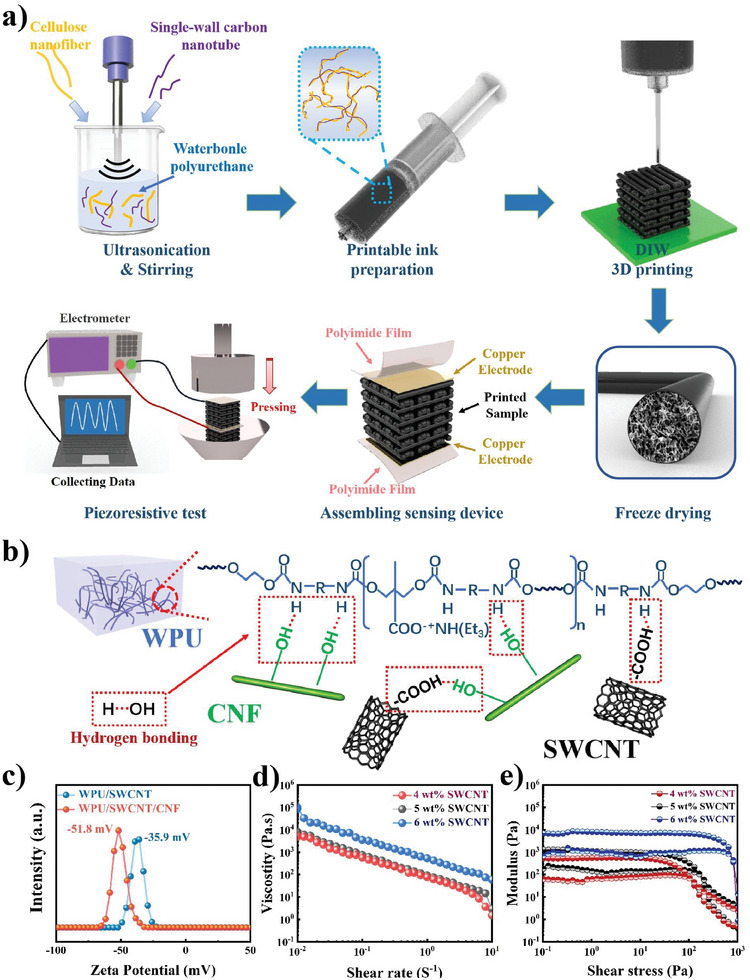
a) Schematic illustrations of the fabrication process of flexible piezoresistive WPU/SWCNT/CNF sponge employing DIW 3D printing consolidated lyophilization strategy, the overall assembly, and the test platform for pressure sensing measurement of sensing device. b) Schematic illustration of the interaction between SWCNT, CNF, and WPU. c) Measurement of the zeta potential of SWCNT/WPU and SWCNT/CNF/WPU suspension. The rheological behavior of WPU/SWCNT/CNF composite ink: d) viscosity as a function of shearing rate; e) the dependence of storage and loss modulus on shear stress.

**Figure** **1**a shows the fabrication process of the sponge employing the DIW printing integrated lyophilization technique. First, a homogeneous composite ink was obtained by mixing single wall carbon nanotubes (SWCNTs), cellulose nanofibril (CNF), and WPU under ultrasonication, followed by dispersion using a high‐speed mixer. The obtained composite ink was extruded from the nozzle to construct arbitrary 3D geometrics via layer‐by‐layer deposition. Subsequently, the printed samples were subjected to lyophilization for water removal and 3D interconnected micropore generation and finally assembled for the sensing device.

The SWCNT loading determined the electrical conductivity of the composite. Before implementing the structural design via 3D printing, the SWCNT dosage was first optimized by employing WPU/SWCNT/CNF microscopic porous composites prepared via drop cast and freeze‐drying. Figure S1 (Supporting Information) shows the electrical conductivity of the composite sponges as a function of SWCNT weight fraction. At SWCNT contents of less than 1 wt. %, the WPU/SWCNT/CNF composite exhibited a relatively weak conductivity of 10^−8^ S m^−1^. This may be because the SWCNTs were not densely packed to enable electron transfer. A significant increase in electrical conductivity occurred as the SWCNT content increased from 1.2 to 5 wt. %, resulting from the well‐established conductive network. In addition, the sponge exhibited percolation behavior. The percolation threshold (φ_c_) can be determined as σ = σ_0_ (φ−φ_c_)^t^ according to the classical percolation theory^[^ ^17^ ^]^ (calculation curve see inset Figure S1, Supporting Information). φ_c_ was calculated as 2.53 wt. %. It has been reported that as a conductive filler near the electrical percolation threshold, the resistance change of the scaffold increases to achieve excellent sensitivity.^[^ ^18^, ^19^ ^]^ In addition, good electrical conductivity is preferred for constructing piezoresistive devices.^[^ ^20^ ^]^ Therefore, SWCNT contains more than 3 wt. % were selected for formulation optimization.

Homogenously dispersed SWCNT in polymer solutions and good affinity between the polymer matrix components are necessary for printable composite inks because they directly affect the mechanical and piezoresistive performance of the printed parts. As shown in Figure 1b, the presence of hydroxyl groups on CNF is conducive to the formation of hydrogen bonds with the bearing of carboxyl moieties on SWCNT and N─H group of WPU. This promotes the interface combination between CNF, SWCNT, and WPU. With the facilitation of negatively charged CNF, SWCNTs are prevented from stacking by strong electrostatic repulsion, resulting in well‐dispersed ink.^[^ ^21^ ^]^ The ink with CNF shows a higher zeta potential of −51.8 mV than that of without CNF at −35.9 mV, indicating that adding CNF is more conducive to the dispersion of SWCNT in ink (Figure 1c).

Ink must be formulated to achieve a specific rheological performance to ensure that DIW 3D printing successfully builds the required architectures. WPU/SWCNT dispersions with various SWCNT weight fractions (4, 5, and 6 wt. % e. g.) demonstrated fluid behavior without any printability (Figure S2, Supporting Information). Upon incorporation of a small amount of cellulose (1.31 wt. %), the ink immediately transformed from fluid to viscous. Typical rheological requirements for printable inks include shear‐thinning behavior, sufficient storage modulus (G’>1000 Pa), and yield stress (τ >200 Pa).^[^ ^22^ ^]^ As shown in Figure 1d, the apparent viscosity of the composite inks with different SWCNT contents (4–6 wt. %) exhibited typical shear‐thinning behavior. Upon shearing, the ink transformed from a highly viscous slurry to a fluid state, addressing the nozzle clogging issue to allow continuous printing under pneumatic pressure. Figure 1f shows the corresponding storage and loss moduli as a function of shear stress for the composite ink containing SWCNT ranging from 4–6 wt. %. The higher plateau of the storage modulus refers to that of the loss modulus, reflecting the behavior of the viscoelastic ink and indicating the possibility of shape retention after programmed deposition.^[^ ^21^ ^]^ As the SWCNT content was increased to 6 wt. %, the storage modulus and yield stress increased to 6.89×10^3^ Pa and 800 Pa, respectively. This satisfies the requirement for sufficient storage modulus and yield stress to better hold the geometries after successful extrusion. As shown in Figure S3 (Supporting Information), the printed lattice based on the 6 wt. % SWCNT retained its shape steadily after being placed in the air for 10 min. Therefore, the ternary composite inks with 6 wt. % SWCNT was selected after a comprehensive investigation of the electrical conductivity and rheological performance.

### 2.2 3D Printing of 3D Interconnected WPU/SWCNT/CNF Derived Sponge

**Figure 2:**
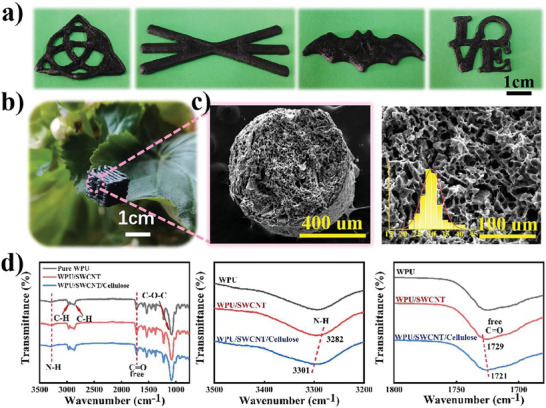
a) Optical images of printed sponges based on WPU/SWCNT/CNF: “special‐shaped pattern”, “furcation”, “bat” and “love” logos in turns. b) A digital image of the printed porous lattice sponge sitting on a leaf. c) The SEM images and aperture distribution of composite sponge. d) FT‐IR spectrums of pure WPU, WPU/SWCNT, and WPU/SWCNT/CNF sponge in the wavenumber range of 800–3500 (left), 3200–3500 (middle) and 1650–1800 cm^−1^ (right).

The desired architectures can be printed directly with formulated WPU/SWCNT/CNF compound inks containing 6 wt% SWCNT. Representative customized structures of “special‐shaped pattern”, “furcation”, “bat”, and “love” logos can be easily constructed (**Figure** **2**a). The size of the printed 3D lattice structure sponge is 10×10×10 mm, with a density of 0.12 g cm^−3^, making it light enough to be stably placed on a leaf (Figure 2b). The scanning electron microscopy (SEM) images with low and high sponge magnifications indicate that the average diameter of the micropores induced by ice crystal removal was ≈ 30 µm (Figure 2c). The soft sponge exhibited superior compression resilience; it can recover to its original shape after being subjected to deformation up to 50% strain (Figure S4, Supporting Information).

The interface investigation between WPU, CNF, and SWCNT was performed using Fourier transform infrared spectroscopy (FT‐IR) (Figure 2d). The FT‐IR spectrum of pure WPU exhibited two typical absorption peaks at 3282 and 1729 cm^−1^, attributed to the N─H stretching vibration and characteristic signal of C═O, respectively. In addition, the peaks at 2790 and 2860 cm^−1^ belong to the asymmetrical and symmetrical stretching absorption bands of C─H in methylene, respectively. After incorporating the SWCNTs, no apparent shift of these characteristic signals occurred, indicating a weak interaction between WPU and SWCNT. However, a clear shift of the N─H signal from 3282 to 3301 cm^−1^ and a blue shift of the C═O peak from 1729 to 1721 cm^−1^ appeared for the WPU/SWCNT/CNF composite sponge owing to the formation of hydrogen bonds between the N and H of WPU and ‐OH of CNF,─COOH of SWCNT, and ─OH of CNF.^[^ ^20^ ^]^ Thus, CNF allows SWCNT to be homogeneously dispersed in the CNF/WPU matrix and has good affinity with WPU. This is highly beneficial for good stress transfer, enhancing the mechanical performance of the sponge.

### 2.3 Mechanical and Piezoresistive Performance of 3D‐Printed WPU/SWCNT/CNF Composite Sponge

To illustrate the advantage of the 3D structure design in enhancing the transduction efficiency of pressure into the resistance signal, a lattice structure and block device (thickness of 9 mm) derived from the WPU/SWCNT/CNF composite were prepared to evaluate the piezoresistive performance. The finite element method (FEM) of COMSOL Multiphysics was adopted to simulate the internal stress and strain distribution of the flat block and lattice structure under an identical external force of 10 N. Figure S5a (Supporting Information) shows the color‐coded results. The lattice can promote the stress accumulation effect. Specifically, the response internal stress distribution was 5.53×10^3^ and 4.71×10^5^ N m^−2^ for the lattice, higher than that of the block device (4.31×10^3^ and 2.54×10^5^ N m^−2^). A larger internal stress typically results from a higher displacement variation, which can be verified by simulating the response strain. As shown in Figure S5b (Supporting Information), the lattice had a high value of 2.39 mm, whereas the value for the block structure was 1.46 mm. This demonstrates the stress–strain amplification effect induced by the reduced modulus of the delicately designed lattice structure, which may further affect the sensitivity performance of the piezoresistive sensor.

**Figure 3:**
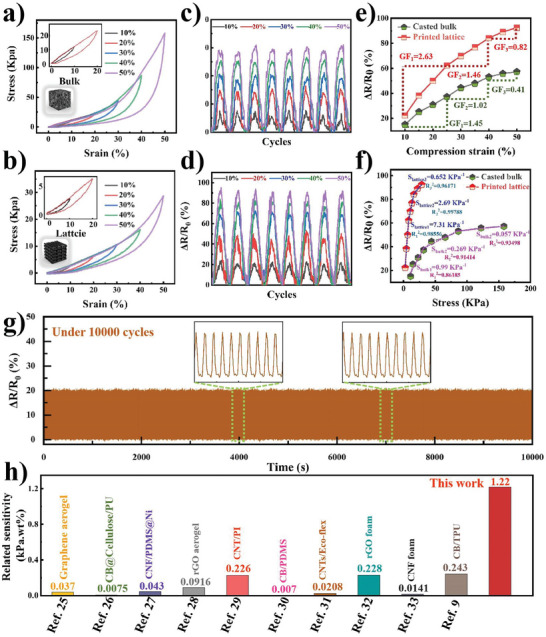
Cyclic compression stress‐strain curves of a) casting bulk and b) printing lattice sponge from 10 to 50%. Multiple cycle tests of relative resistance under progressively increased stain of c) block and d) printed 3D latticed sponge. Relative resistance as a function of e) compression strain and f) applied stress of flat block and printed 3D latticed sponge. g) Durability test of the 3D printed lattice sponge at compression strain of 10% for 10 000 working cycles h) Comparison of relative sensitivity between this work and the mainstream work of carbon‐based conductive composite aerogel/foam derived piezoresistive sensors.

The compression properties of devices play are significant for practical applications. A series of cyclic compression tests at different compression strains (10%, 20%, 30%, 40%, and 50%) were performed on composite blocks and lattices. **Figure** **3**a,b shows the corresponding compressive stress–strain curves. The coincidence degree of the curves was offset when the block was compressed to 50%. This indicates that irreversible deformation occurred at this time. However, when the lattice structure was continuously compressed to 50%, its curve coincidence degree was high, indicating that it could maintain excellent compression recovery under this compressive strain. In addition, the modulus of the lattice (0.19 kPa) was lower than that of the block (1.11 kPa) because the programmed macroscopic holes favor the stress amplification effect, which is consistent with the above simulation results. In addition, long‐term compression stability tests were performed to evaluate the durability of the devices over 10 000 cycles at 50% strain. As shown in Figure S6 (Supporting Information), the composite lattice exhibited an exceptional shape recovery performance with a reversible strain rate of 90.35% at the 10000th cycle for the 50% cyclic compression test. By contrast, the respective rate for the block was only 64.2%, indicating that proper structure design can reduce energy dissipation.^[^ ^23^ ^]^

Figure 3c,d shows the relative resistances (ΔR/ΔR_0_ = (R_0_‐R)/R_0_, where R_0_ and R represent the resistance of the piezoresistive device at the initial state and under compression, respectively) of the WPU/SWCNT/CNF composite and block and lattice sponge devices under progressively increasing strain amplitudes. The variation in relative resistance synchronously increased with increasing strain amplitude for both block and lattice devices when tested at a wide range of compression strains (ε = 10%, 20%, 30%, 40%, and 50%), suggesting that a larger strain enables more significant conductive network alteration and typical piezoresistive sensing behavior. Figure 3e shows the dependence of the relative resistance on the applied compressive strain and the gauge factor (GF = (ΔR/R_0_)/ε, where ε represents the respective strain) of the lattice and block. The printed lattice device exhibited a significantly higher relative resistance than that of the block device. The lattice structure exhibited gauge factors of 2.63, 1.46, and 0.82 at 10–25%, 25–40%, and 40–50% strain ranges, respectively. The highest GF for the block device was 1.45, 80% lower than that of the lattice at 10–25% strain. The apparent enhancement in the GF of the lattice structure is attributed to the presence of many connection points between the filament, where stress concentration occurs upon compression. In addition, connection points exist on all paths through which the current passes, which is equal to the series resistors. Thus, compression deformation in a 3D lattice structure can increase the resistance variation, enhancing the gauge factor.^[^ ^24^ ^]^

In addition, the sensitivity of the proposed composite lattice and block structures to the applied pressure was also studied. The pressure sensitivity S = (ΔR/ΔR_0_)/σ is the resistance change ratio divided by the compressive stress. Figure 3f shows the pressure response behavior, expressed as the dependence of the relative resistance on the applied pressure. The relative resistance exhibited a linear increase with increasing pressure. The lattice structure demonstrated sensitivity slopes of 7.31, 2.69, and 0.652 kPa^−1^ at 2.8–8.1, 8.1–16.2, and 16.2–28.7 kPa stress ranges, respectively. The highest sensitivity slope of the block was 0.99 kPa^−1^, ≈ 7.3 times lower than that of the lattice architecture. The substantial increase in sensitivity can be associated with the amplified stress–strain effect of the lattice structure, producing more contact area and conductive paths and enhancing the resistance variation upon the applied compression stress. Therefore, it can be concluded that the sensitivity of PPS can be improved by the rational structural design of conductive materials to enhance their resistance change by reducing their mechanical strength and modulus while retaining elasticity. In addition, the latticed piezoresistive sensor was examined through a cyclic loading test (1 Hz) for 10 000 continuous working cycles (Figure 3g). It can be clearly seen that there is no significant attenuation of the resistance signal throughout the cycle, suggesting superior mechanical durability.

A comprehensive comparison of the piezoresistive performance of the 3D‐printed WPU/SWCNT/CNF proposed composite lattice sponge with the reported carbon‐based composites was conducted to illustrate the advancement of the proposed method. As summarised in Figure 3h; Table S1 (Supporting Information), the as‐designed sensor exhibited the highest relative sensitivity of 1.22 (kPa^*^wt. %)^−1^ under a pressure range of 2.8–8.1 kPa, approximately five times that of a state‐of‐the‐art carbon‐based conductive composite aerogel/foam‐derived piezoresistive pressure sensor.^[^ ^25^, ^26^, ^27^, ^28^, ^29^, ^30^, ^31^, ^32^, ^33^ ^]^ This advantage was primarily attributed to the stress amplification effect from the hierarchically porous sponge structure.

### 2.4 3D Printing of Customized Sponges for Tunable Anisotropy and Directional Response

**Figure 4:**
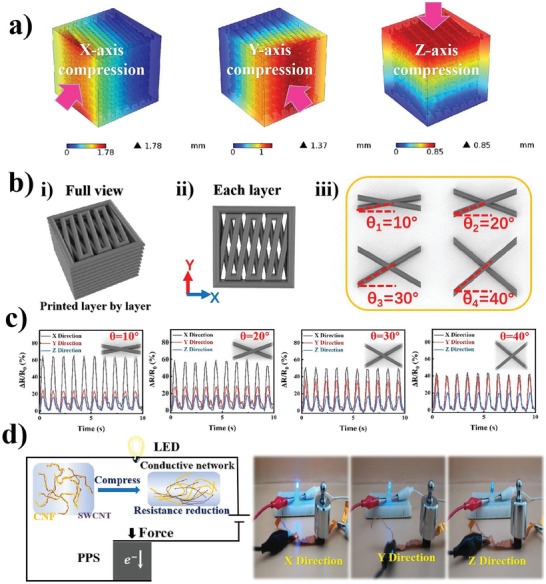
a) Simulation results of strain distribution for anisotropic lattice structure under identical external force at X, Y, and Z axis. b) Schematic of the model design for four cross structures. c) The response relative resistance of four designated “scissor‐like” structures under identical periodic impact coming from X, Y, and Z directions. d) Circuit diagram and photographs of the anisotropic lattice (θ = 30°) connected with an LED lamp to visualize the brightness change under the same weight in X, Y, and Z directions.

In addition to the printed WPU/SWCNT/CNF composite lattice featuring excellent mechanical and sensing performance, structural design was applied to fully control the directional response by adjusting the spatial arrangement of piezoresistive filaments. The anisotropy of the designated lattice with cross‐unit “scissors‐like” structures was predicted through FEM using the COMSOL Multiphysics software package. The internal stress distribution and response displacement variation of the lattice structure in the three principal directions under identical external compression forces were simulated. In the FEM model, the simulated size of the designed 3D lattice was set to 6 mm × 6 mm × 6 mm. The bottom of the cell was fixed, and the force was exerted along the normal direction. Figure S7 (Supporting Information) shows the detailed boundary‐setting conditions and model meshing modes. As shown in Figure S8 (Supporting Information), the simulated response internal stress distribution changed with the exerted force direction, with values of 1.35×10^3^–2.69×10^5^, 822–2.27×10^5^, and 2.83×10^3^–1.48×10^5^ N m^−2^ under compression in the X, Y, and Z directions, respectively. A larger internal stress distribution indicates a better stress amplification effect, resulting in more phenomenon strain; therefore, the displacement trend is consistent with the stress distribution. As shown in **Figure** **4**a, the simulation of the response displacement variation showed distinct values at different force directions, which are 1.78, 1.37, and 0.85 mm in the X, Y, and Z directions, respectively. The different magnitudes of strain will inevitably affect the lap and fracture of the conductive path upon compression. This causes differentiation in the relative resistance, leading to different sensitivities in the three orthogonal directions. The simulation results suggest that the lattice featuring cross‐unit structures has anisotropic characteristics in terms of sensitivity.

Anisotropy can be tuned by adjusting the printing scheme. The proposed strategy is initiated by changing the stacking angle (θ) of the filaments between the neighboring layers. Four lattices were printed with θ of 10°, 20°, 30°, and 40° (Figure 4b). **T**he relative resistance (Figure 4c) and stability (Figure S9, Supporting Information) under periodic exertion of an external pressure (5 kPa) are tested. The moment arm of the “scissors‐like” structure along the X‐axis was larger when θ was smaller (for example, when θ = 10°). Moreover, the compressive strain was the largest, resulting in the largest resistance variation. By contrast, the moment arm along the Y‐axis was the shortest owing to the geometric relationship of the vertical angle; thus, the degree of compression was the smallest, resulting in the smallest resistance change. Therefore, the “scissor‐like” 3D grid structure exhibited significant and controllable resistance changes in the X and Y directions. In addition, the number of layers was consistent with the height because the four structures were stacked and printed layer‐by‐layer upward in the Z‐axis direction. In addition, the enhancement advantage brought by the “scissor‐like” structure was not highlighted; thus, there was no apparent difference in the degree of resistance variation. The resistance response in three directions under compression strain was detected and calculated for GF (Figure. S10, Supporting Information) to quantify the degree of sensitivity of anisotropic structures in different directions. Consequently, the relative resistance varied along each direction for the four printed architectures. Particularly for the lattice with θ = 30°, the device exhibited the most apparent differentiation in the degree of relative resistance variation, corresponding to GF values of 2.485, 3.085, and 1.835 in the X, Y, and Z directions, respectively.

A lattice with θ = 30° was connected to a closed circuit to visualize the real effect caused by anisotropic capability. The PPS can be regarded as a resistive element in a circuit whose resistance decreases as the pressure increases. Compression by an identical force along different directions lit up the LED lamp to a distinct brightness. Figure 4d and video S1 (Supporting Information) show the highest brightness in the X‐direction and the lowest brightness in the Z‐direction, whereas the Y‐direction remained in‐between. This was consistent with the sensitivity performance. This simple example indicates the potential that 3D printing‐empowered structure customization can pave the way for multifunctional piezoresistive sensing systems.

### 2.5 Applications of Skin‐Inspired Flexible Pressure Sensor

**Figure 5:**
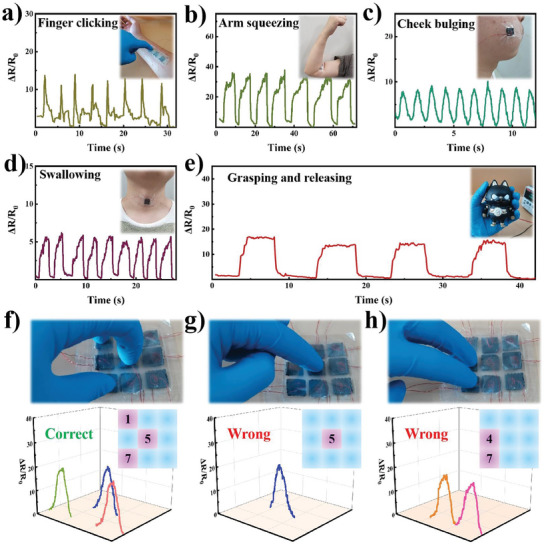
Wearable device assembled from WPU/SWCNT/CNF derived sponge as an active layer. a) relative resistance signals caused by finger clicking, b) arm squeezing, c) cheek bulging, d) swallowing. e) Relative resistance change monitored by the sponge sensor attached to the fingertip upon grasping and releasing of a solid toy cat. Intelligent combination lock integrated by 9 sensors reflected its f) correct and g), h) incorrect passcodes for decoding the lock.

Owing to the piezoresistive performance of the latticed sponge device, the as‐fabricated device was assembled to explore its potential applications in robotic skins. As shown in **Figure** **5**a,d, distinct signals were obtained upon finger clicking, arm squeezing, cheek bulging, and swallowing, indicating the capability of force sensing with different magnitudes. In addition, a piezoresistive tactile sensor was attached to the fingertip to monitor the gripping force in real‐time, as shown in Figure S11 and Video S2 (Supporting Information), indicating a rapid response to external stimuli. A solid toy cat underwent periodic grasping and release, and the corresponding response relative to resistance variation was recorded (Figure 5e). The shape of each cycle can be classified into three stages: 1) the quasi‐linear growth period reflecting the gradual increase in the exerted force during grasping; 2) the nearly plateau area, indicating the holding of the toy; and 3) the steep signal reduction correlating to the force‐releasing stage. Therefore, an intelligent combination lock integrated by nine piezoresistive sensors was demonstrated. Each sensor was labeled “1” to “9” in sequence. The sensor was pressed at the as‐setting position, and the resistance change signal was obtained as the password. Figure 5f shows a correct example of when sensors in positions “1”, “5”, and “7” must be pressed at the same time to decode the lock. Only pressing a sensor in position “5” (Figure 5g) or pressing only two sensors in positions “4” and “7” (Figure 5h), are all regarded as incorrectly passed words. The successful demonstration of a tactile sensor indicates attractive application potential in robotic skin monitoring and human‐machine interaction and artificial intelligence.

## 3 Conclusion

This study is the first to develop a flexible 3D PPS with macroscopic 3D structures and microscopic pore morphologies through DIW 3D printing using a home‐made ink comprising an elastomeric conductive composite based on WPU/SWCNT/CNF. The as‐fabricated PPS with a customized anisotropic “scissor‐like” structure exhibited an apparent directional recognition ability, whose anisotropy can be easily tuned by adjusting the printing scheme. Furthermore, the sculptured 3D PPS delivered a high relative sensitivity over a broad pressure range (2.8–8.1 kPa), surpassing that of a state‐of‐the‐art carbon‐based conductive composite aerogel/foam‐derived PPS.

## 4 Experimental Section

### Materials

Carboxylated SWCNTs with a length of 5–30 µm were obtained from XF Nano Co. with an electrical conductivity of 1.75×10^6 ^S m^−1^. Sodium hypophosphite (SHP) and 1,2,3,4‐butanetetracarboxylic acid (BTCA) and conductive silver paste were purchased from Aladdin, Inc. CNF powder was obtained from Guilin Cellulose Technology Co. Commercially available WPU with a solid content of 30 wt. % was purchased from Hefei Hengtian New Material Technology Co.

### Preparation of DIW Ink

The functional ink comprised SWCNT, CNF, BTCA, SHP, and WPU. First, the SWCNT, CNF, WPU, and deionized water in the designated mass ratio were subjected to ultrasonication to obtain a composite slurry (mass ratio of CNF: WPU: BTCA: SHP = 1:22:0.3:0.3, the SWCNT content was defined as 4, 5, and 6 wt. % of the solute mass for different formulations) and dispersed by a high‐speed homogenizer (IKA T10 ULTRA‐TURRAX) for 30 min to obtain a stable dispersion.

### 3D Printing of Lattice Sponges

A three‐axis motion stage with a programmable robotic dispenser (Allevi 3 Bioprinter, USA) was used to print the formulated ink on a glass slide substrate at a speed of 10 mm−^1 ^s to construct the designated structures. The ink was loaded into a 20 CC dispensing syringe with a 0.8 mm diameter micronozzle and extruded by an air‐powered fluid dispenser at a pressure range of 6–20 psi, depending on the mass fraction of SWCNT fillers. The printed parts were subjected to liquid nitrogen and lyophilization to obtain the composite sponges. Finally, the sponges were placed in an oven at 170 °C for 45 min, enabling the SHP catalyzed cross‐linking between BTCA and CNF to improve the mechanical robustness of the matrix.

### Characterisation

The ink rheology in shear viscometry and oscillatory modes was assessed using a rheometer (TA Instruments Company, AR2000EX). The apparent viscosity was obtained as a function of shear rate (0.01–10 s^−1^), and the storage and loss moduli were measured versus shear stress (1–1000 Pa) at a fixed frequency of 1 Hz. Fourier transform infrared spectroscopy (FT‐IR) spectra in the range of 500–4000 cm^−1^ were collected on a Nicolet Nexus 870 instrument at a resolution of 4 cm^−1^ using the attenuated total reflection mode. A four‐probe tester was used to measure the conductivity of the sponge. The microstructures of the prepared samples were characterized using scanning electron microscopy (SEM, Philips Company, FEI Nova 400). Mechanical compression strain/stress experiments were performed using an Instron 5567 machine (USA) at room temperature. The resistance signals of the samples were measured in real‐time using a Keithley 2026 B source meter (USA).

### Piezoresistive Measurement

The upper and lower surfaces of the sample were coated with silver paste and affixed to copper foil electrodes. A wire was then drawn from each electrode to connect to the source meter for testing under mechanical compression. The experiments were conducted at a relative humidity of 40% ± 5% and room temperature.

## Conflict of Interest

The authors declare no conflict of interest.

## Supporting information

Supporting Information

Supplemental Video 1

Supplemental Video 2

## Data Availability

The data that support the findings of this study are available from the corresponding author upon reasonable request.
