# Supplementary material for: 3D Printing of Anisotropic Piezoresistive Pressure Sensors for Directional Force Perception
Source: Adv Sci (Weinh). 2024 Mar 13;11(24):2309607. doi: 10.1002/advs.202309607 (PMC11199969; doi:10.1002/advs.202309607)
Supplement: Supplementary file 1 — Supporting Information [file ADVS-11-2309607-s003.pdf]

ADVANCED SCIENCE
Open Access

Supporting Information

for *Adv. Sci.*, DOI 10.1002/advs.202309607

3D Printing of Anisotropic Piezoresistive Pressure Sensors for Directional Force Perception

Jingfeng Liu, Xuan Zhang, Jintao Liu, Xingang Liu and Chuhong Zhang**

Supporting information

Three-dimensional Printing of Anisotropic Piezoresistive Pressure Sensors for Directional Force Perception

Jingfeng Liu, Xuan Zhang, Jintao Liu, Xingang Liu, Chuhong Zhang**

State Key Laboratory of Polymer Materials Engineering, Polymer Research Institute of Sichuan University, Chengdu, 610065, China

E-mail: liuxingang@scu.edu.cn, chuhong.zhang@scu.edu.cn

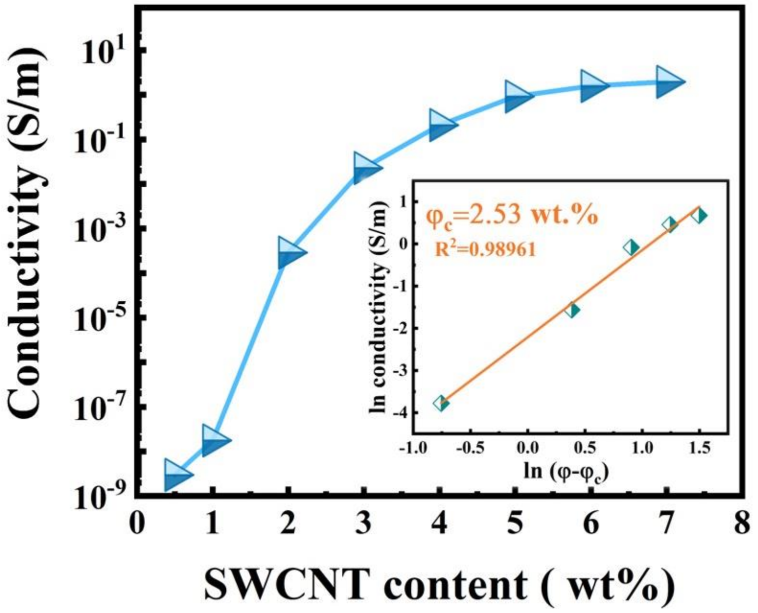

Figure S1. Electrical conductivity of composite aerogel with varied SWCNT content. the insert is the fitting of experimental data based on the percolation theory.

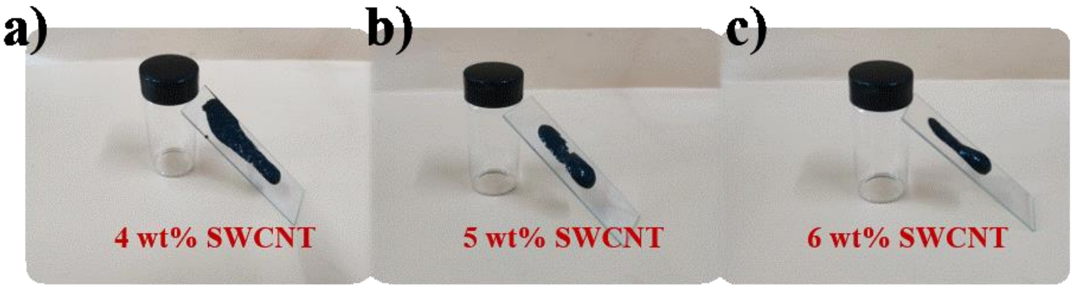

Figure S2. Image of the WPU dispersion containing a) 4 wt.%, b) 5 wt.% SWCNT and 6 wt.% SWCNT.

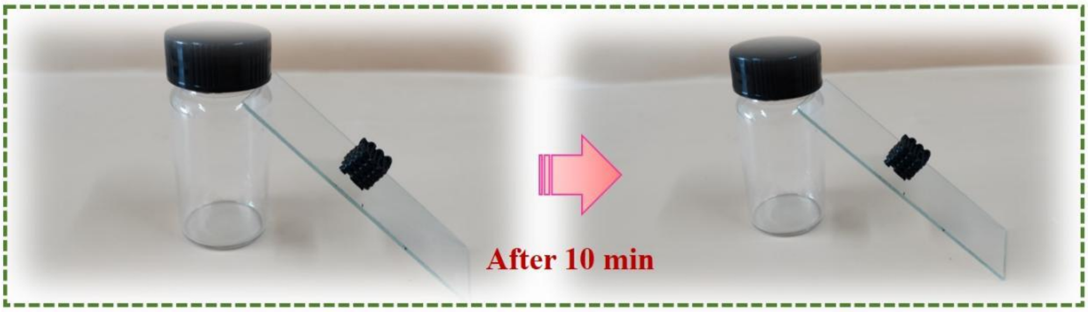

Figure S3 The printed WPU/SWCNT/CNF ink with 6 wt.% SWCNT.

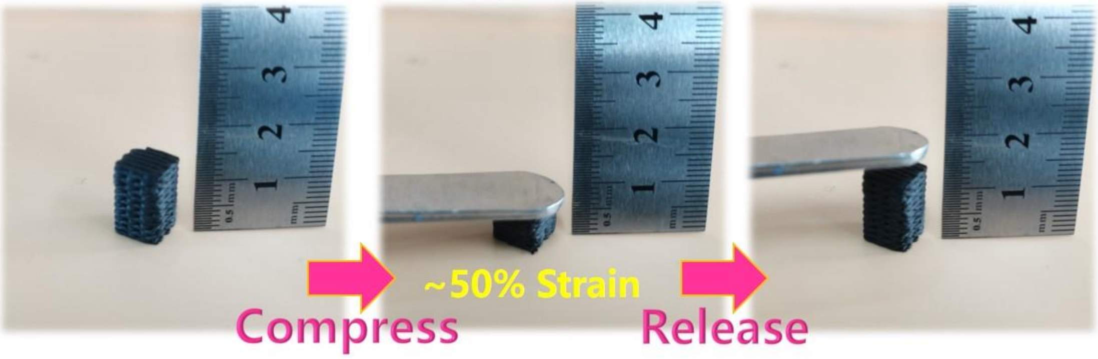

Figure S4. Sponge appearance under compression and after releasing.

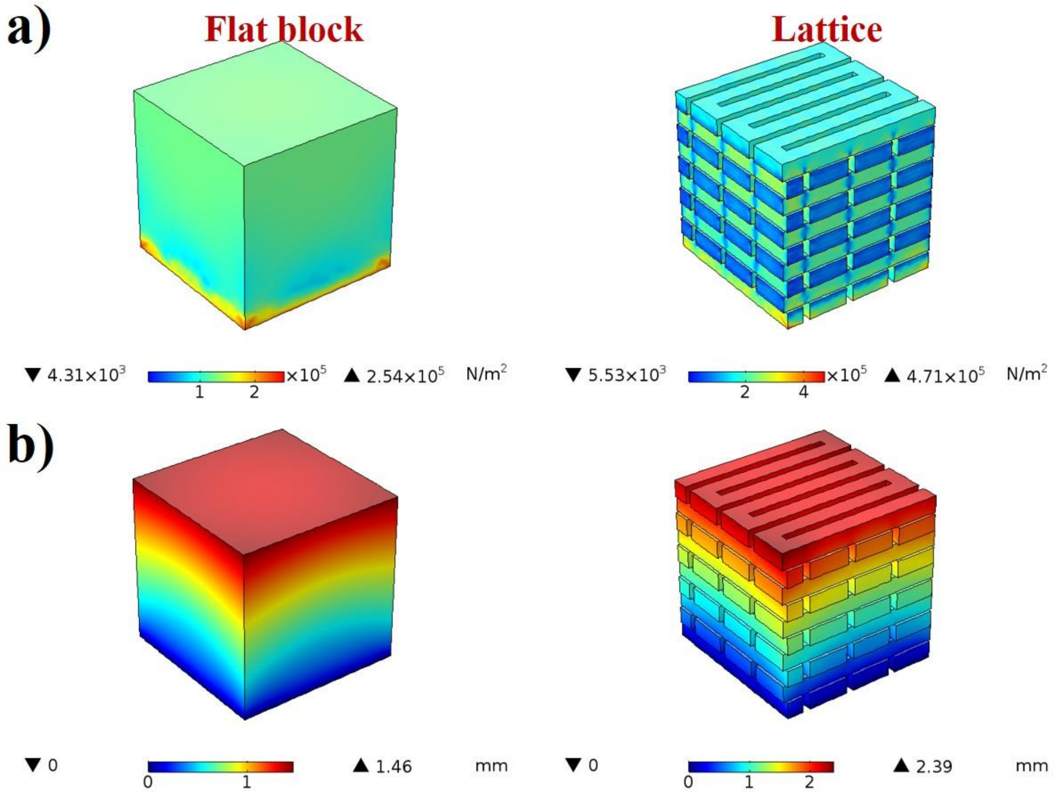

Figure S5. Simulation results of the stress distribution (a) and strain variation (b) for block and lattice structure under the identical external force of 10 N.

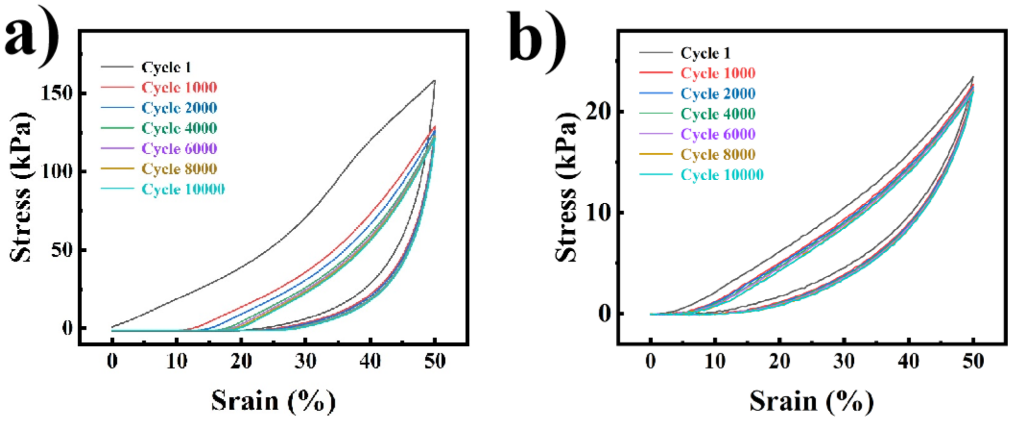

Figure S6. Long-term stability test of the a) flat block and b) lattice structure during 10000 cycles under 50% compression strain.

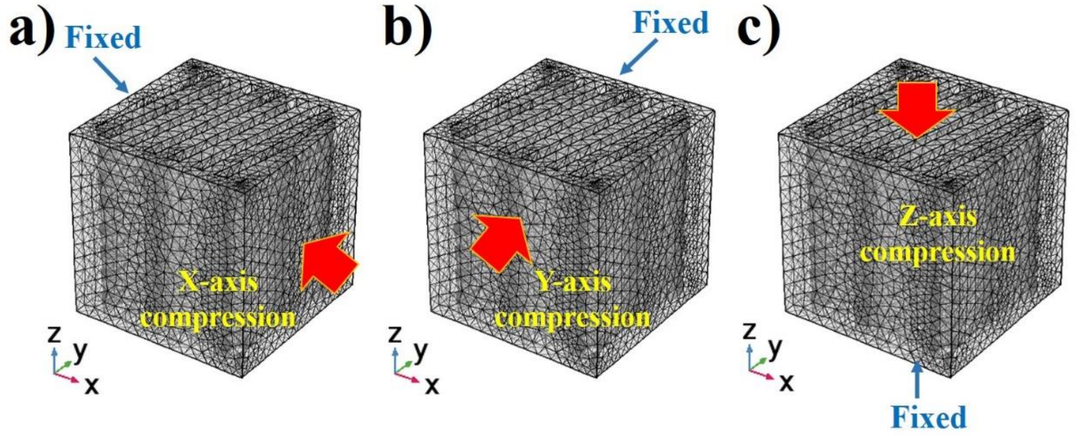

Figure S7. The meshing results of designed cross structure and the boundary conditions under a) X direction, b) Y direction and c) Z direction.

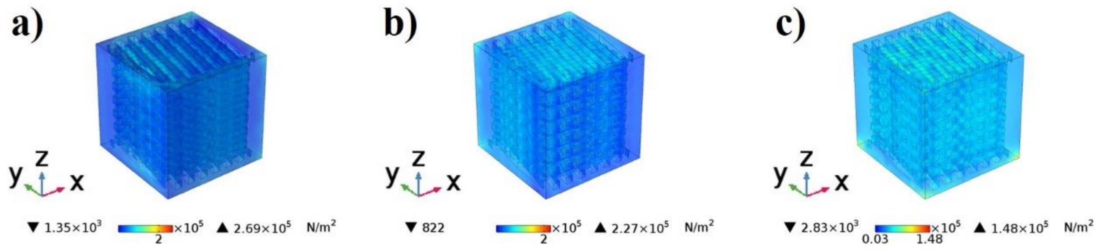

Figure S8. Simulation results of the stress distribution of the anisotropic lattices (6×6 ×6 mm) under identical external force of 5 N with force direction of a) X-direction, b) Y-diretion and c) Z-direction.

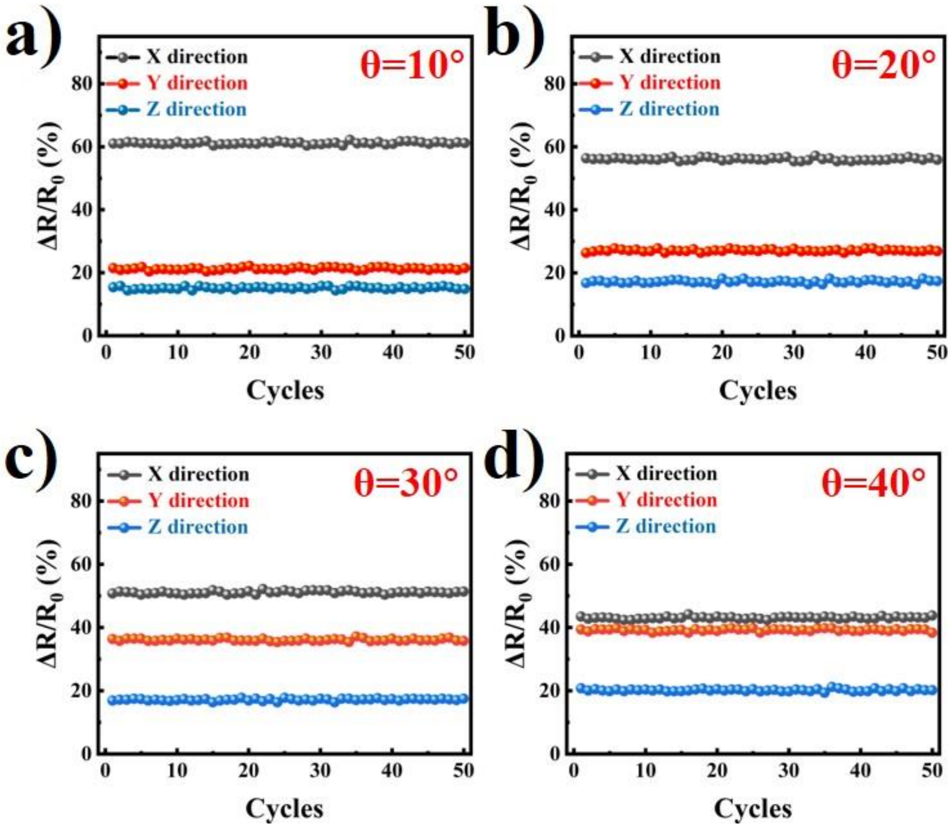

Figure S9. The response relative resistance of four designated “scissor-like” structures with a) θ=10°, b) θ=20°, c) θ=30°, d) θ=40°under long-term periodic impact coming from X, Y and Z direction.

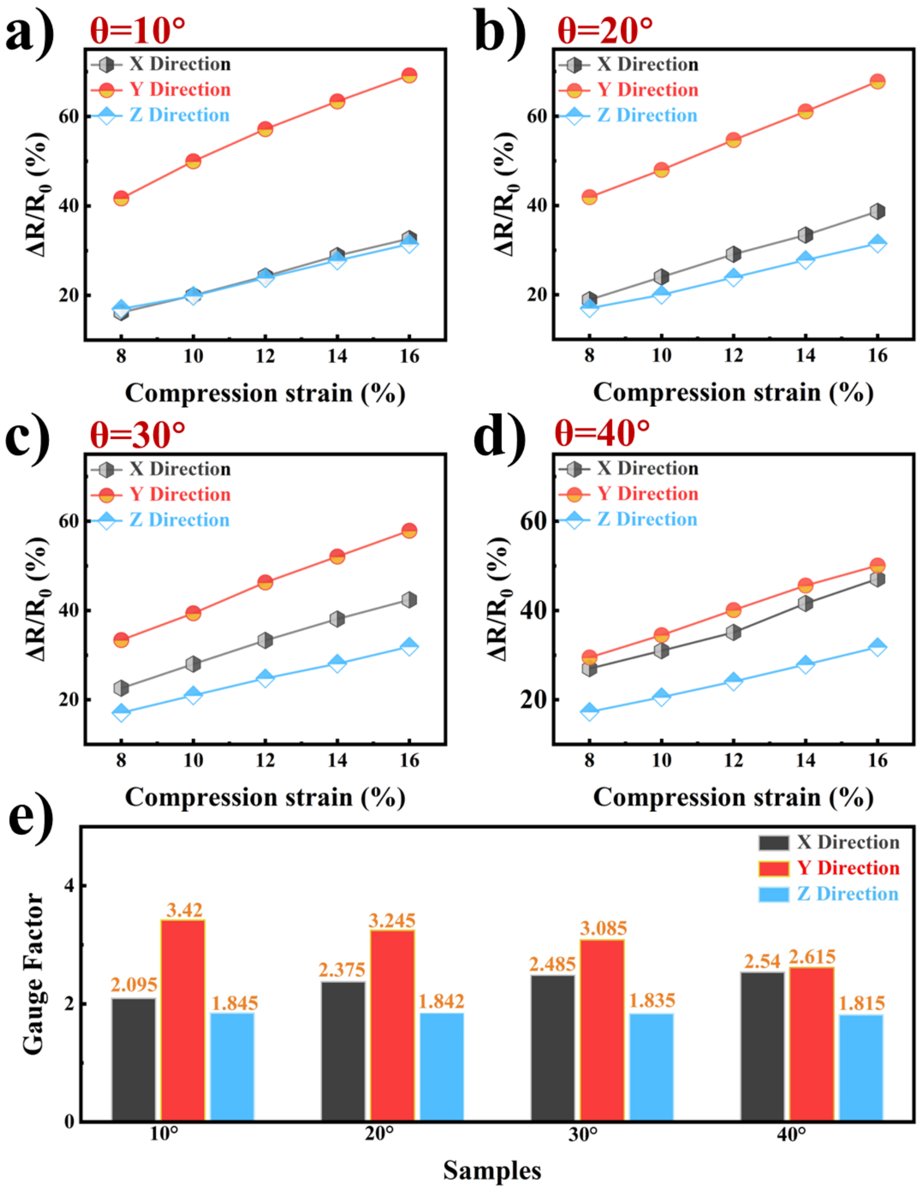

Figure S10. The response relative resistance changes of four designated “scissor-like” structures with a) θ=10°, b) θ=20°, c) θ=30°, d) θ=40° under continuous compression strains (from 8% to 16%), and the e) calculation of GF values, on X, Y, and Z directions.

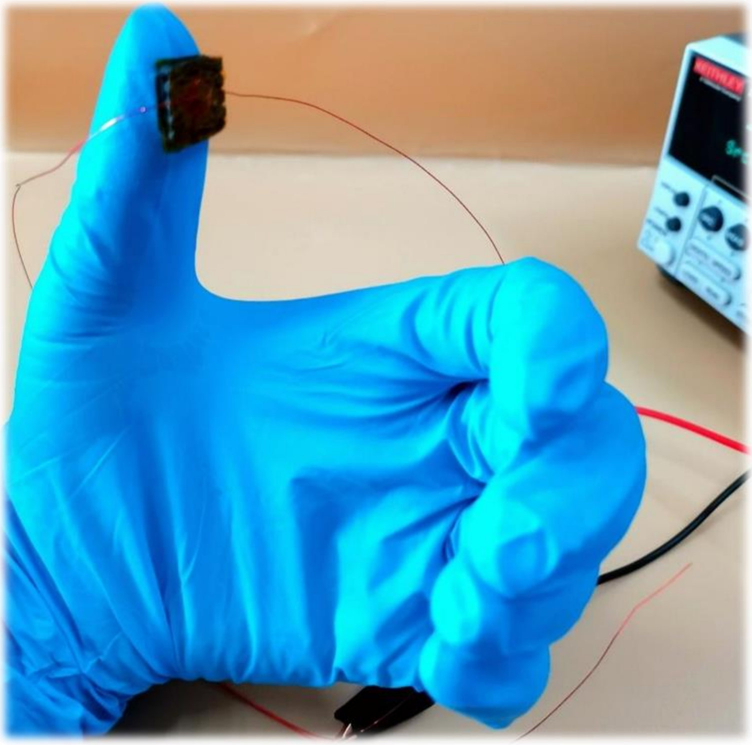

Figure S11. Photograph of the printed sponge pressure sensor assembled on the fingertip of a nitrile glove.

Table S1. Piezoresistive pressure sensors based on carbon-based elastomeric composite derived aerogel and foam.

Material	Sensitivity (kPa^{-1})	Load range (kPa)	Filler content (wt.%)	Relative sensitivity $(kPa.wt\%)^{-1}$	Ref.
Graphene aerogel	3.69	1.5×10^{-4}~0.6	~100	0.037	[1]
CB@Cellulose/PU	0.35	0~29	46.9	0.0075	[2]
CNF/PDMS@Ni	0.6	0~20	14	0.043	[3]
rGO aerogel	9.13	1.43~13.56	~100	0.091	[4]
CNT/PI	11.28	0~61	50	0.226	[5]
CB/PDMS	0.014	0~100	2	0.007	[6]
CNTs/Eco-flex	0.096	0~175	4.6	0.0208	[7]
rGO foam	22.8	0.01~0.1	~100	0.228	[8]
CNF foam	1.41	0~2.5	~100	0.0141	[9]
CB/TPU	5.5	0~440	22.6	0.243	[10]
SWCNT/CNF/WPU	7.31	2.8~28.7	6	1.22	This work

1D/2D Nanomaterials Synergistic, Compressible, and Response Rapidly 3D Graphene Aerogel for Piezoresistive Sensor

Reference:

[1] P. Min, X. Li, P. Liu, J. Liu, X.-Q. Jia, X.-P. Li, Z.-Z. Yu, *Adv. Funct. Mater.* 2021, **31**, 2103703.

[2] S. Xu, X. Li, G. Sui, R. Du, Q. Zhang, Q. Fu, *Chem. Eng. J.* 2020, **381**, 122666.

[3] S.-W. Dai, Y.-L. Gu, L. Zhao, W. Zhang, C.-H. Gao, Y.-X. Wu, S.-C. Shen, C. Zhang, T.-T. Kong, Y.-T. Li, L.-X. Gong, G.-D. Zhang, L.-C. Tang, *Comp. Part B: Eng.* 2021, **225**, 109243.

[4] J. Huang, J. Zeng, B. Liang, J. Wu, T. Li, Q. Li, F. Feng, Q. Feng, M. J. Rood, Z.

Yan, *ACS Appl. Mater. Interfaces* 2020, **12**, 16822.

[5] X. Chen, H. Liu, Y. Zheng, Y. Zhai, X. Liu, C. Liu, L. Mi, Z. Guo, C. Shen, *ACS Appl. Mater. Interfaces* 2019, **11**, 42594.

[6] Y.-F. Wang, T. Sekine, Y. Takeda, J. Hong, A. Yoshida, D. Kumaki, T. Shiba, S. Tokito, *Adv. Mater. Technol.* 2021, **6**, 2100731.

[7] Z. Tang, S. Jia, C. Zhou, B. Li, *ACS Appl. Mater. Interfaces* 2020, **12**, 28669.

[8] X. Zang, X. Wang, Z. Yang, X. Wang, R. Li, J. Chen, J. Ji, M. Xue, *Nanoscale* 2017, **9**, 19346.

[9] Z. Han, Z. Cheng, Y. Chen, B. Li, Z. Liang, H. Li, Y. Ma, X. Feng, *Nanoscale* 2019, **11**, 5942.
